# Cognitive and social activities and long-term dementia risk: the prospective UK Million Women Study

**DOI:** 10.1016/S2468-2667(20)30284-X

**Published:** 2021-01-28

**Authors:** Sarah Floud, Angela Balkwill, Siân Sweetland, Anna Brown, Elsa Mauricio Reus, Albert Hofman, Deborah Blacker, Mika Kivimaki, Jane Green, Richard Peto, Gillian K Reeves, Valerie Beral

**Affiliations:** a Cancer Epidemiology Unit, University of Oxford, Oxford, UK; b Nuffield Department of Population Health, University of Oxford, Oxford, UK; c Division of Cardiovascular Medicine, University of Oxford, Oxford, UK; d Department of Epidemiology, Harvard T H Chan School of Public Health, Boston, MA, USA; e Department of Psychiatry, Massachusetts General Hospital and Harvard Medical School, Boston, MA, USA; f Department of Epidemiology and Public Health, University College London, London, UK

## Abstract

**Background:**

Although dementia is associated with non-participation in cognitive and social activities, this association might merely reflect the consequences of dementia, rather than any direct effect of non-participation on the subsequent incidence of dementia. Because of the slowness with which dementia can develop, unbiased assessment of any such direct effects must relate non-participation in such activities to dementia detection rates many years later. Prospective studies with long-term follow-up can help achieve this by analysing separately the first and second decade of follow-up. We report such analyses of a large, 20-year study.

**Methods:**

The UK Million Women Study is a population-based prospective study of 1·3 million women invited for National Health Service (NHS) breast cancer screening in median year 1998 (IQR 1997–1999). In median year 2001 (IQR 2001–2003), women were asked about participation in adult education, groups for art, craft, or music, and voluntary work, and in median year 2006 (IQR 2006–2006), they were asked about reading. All participants were followed up through electronic linkage to NHS records of hospital admission with mention of dementia, the first mention of which was the main outcome. Comparing non-participation with participation in a particular activity, we used Cox regression to assess fully adjusted dementia risk ratios (RRs) during 0–4, 5–9, and 10 or more years, after information on that activity was obtained.

**Findings:**

In 2001, 851 307 women with a mean age of 60 years (SD 5) provided information on participation in adult education, groups for art, craft, or music, and voluntary work. After 10 years, only 9591 (1%) had been lost to follow-up and 789 339 (93%) remained alive with no recorded dementia. Follow-up was for a mean of 16 years (SD 3), during which 31 187 (4%) had at least one hospital admission with mention of dementia, including 25 636 (3%) with a hospital admission with dementia mentioned for the first time 10 years or more after follow-up began. Non-participation in cognitive or social activities was associated with higher relative risks of dementia detection only during the first decade after participation was recorded. During the second decade, there was little association. This was true for non-participation in adult education (RR 1·04, 99% CI 0·98–1·09), in groups for art, craft, or music (RR 1·04, 0·99–1·09), in voluntary work (RR 0·96, 0·92–1·00), or in any of these three (RR 0·99, 0·95–1·03). In 2006, 655 118 women provided information on reading. For non-reading versus any reading, there were similar associations with dementia, again with strong attenuation over time since reading was recorded, but longer follow-up is needed to assess this reliably.

**Interpretation:**

Life has to be lived forwards, but can be understood only backwards. Long before dementia is diagnosed, there is a progressive reduction in various mental and physical activities, but this is chiefly because its gradual onset causes inactivity and not because inactivity causes dementia.

**Funding:**

UK Medical Research Council, Cancer Research UK.

## Introduction

Participation in activities that are cognitively stimulating is recommended in national and international guidelines as a way of preventing dementia, particularly for older people.^1^, ^2^, ^3^, ^4^ A recent systematic review^5^ of epidemiological studies concluded that engaging in cognitive and social activities, defined as “popular leisure activities considered to require information seeking or processing”, was associated with a lower incidence of dementia (either Alzheimer's disease or any dementia), and that more frequent participation in such activities was associated with a lower incidence of the disease. However, most of the prospective studies in that review (or published since) had relatively short follow-up periods. Only five studies, all small, had more than 10 years of follow-up.^6^, ^7^, ^8^, ^9^, ^10^ Yet, the pathological processes that culminate in dementia, as indicated by deposition of amyloid and tau^11^ or by development of other cerebral lesions,^12^ tend to be gradual, with preclinical effects that develop only slowly over some years. Consistent with the slow progression of these pathological processes, weight loss and physical inactivity have been reported at least a decade before any definite record of dementia.^13^, ^14^, ^15^, ^16^ The gradual preclinical development of dementia over several years could progressively reduce not only physical but also mental activity, reducing participation in cognitively stimulating activities. Hence, the association between not engaging in such activities and the incidence of dementia during the next few years could reflect inactivity being a consequence rather than a cause of the gradual onset of disease.

**Research in context**

**Evidence before this study**

On March 3, 2020, we searched PubMed for English-language reports of prospective studies with terms related to cognitive and social activities (eg, “cognitive activities”, “leisure activities”, “intellectual activities”, “social participation”) and dementia. For cognitive activities we also used a 2016 systematic review, so our search was only from July, 2014, but for social activities our search had no time limit. Most prospective studies had less than 10 years of follow-up, and although non-participation in cognitive or social activities was associated with dementia, these short-term associations might reflect the consequences of dementia rather than a direct effect of the activities. Investigating this association requires looking backwards at activities many years previously, because of the slowness with which dementia can develop. Prospective studies with long-term follow-up can help achieve this by considering associations separately for various time intervals between the recording of dementia and the recording of non-participation. The strengths of the associations during different time intervals can help assess whether, particularly with shorter follow-up, there are only the expected effects of dementia or incipient dementia on non-participation. Just two prospective studies with follow-up for more than a decade have reported results separately by follow-up time, and although both were small, they suggested that associations found with shorter follow-up became null or weakened with longer follow-up. We report analyses of a large, 20-year prospective study.

**Added value of this study**

This analysis of about 850 000 Million Women Study participants who had reported various social and cognitive behaviours has virtually complete long-term follow-up and linkage to the first detection of dementia in hospital records. Having a mean follow-up of 16 years after participation was recorded meant that we were able to describe reliably dementia detection risks separately in the first and second decade between dementia detection and the recording of cognitive or social activities. Non-participation in these activities was (as in other studies) strongly associated with dementia detection risk during the first 5 years of follow-up and was moderately associated with dementia detection risk during follow-up years 5–9, but had little or no association with dementia detection risk during the second decade of follow-up. The associations found during the first decade together with the null associations during the second decade are similar to associations observed for other activities, such as physical inactivity. Taken together, they provide strong evidence that early manifestations of the disease progressively reduce participation in various mental and physical activities over a period of several years before dementia gets mentioned in any hospital records, and that participation in such activities has little or no effect on the incidence of dementia.

**Implications of all the available evidence**

The available evidence suggests that, long before dementia is diagnosed, there is a progressive reduction in various mental and physical activities, but this is chiefly because the gradual onset of dementia causes inactivity, not because inactivity causes dementia.

To help distinguish between these alternatives, prospective studies are needed with durations of follow-up that are long enough to allow reliable examination of both short-term and long-term associations. Within such studies, biases due to the effects of reverse causality would be greater during the first decade (particularly the first 5 years) after cognitive and social activities were recorded than during the second decade of follow-up. In the 20-year UK Million Women Study,^15^ which began in 1996–2001, about 850 000 participants provided information in 2001 about their engagement in various cognitive and social activities and about 680 000 provided information in 2006 about reading. This information was related to the first record of dementia in a hospital admission (generally for another condition) during the follow-up periods of 0–4 years, 5–9 years, and 10 or more years after the activity was reported. We aimed to use these data to examine the prospective associations between non-participation in adult education, groups for arts, crafts, or music, voluntary work, and reading with the risk of dementia diagnosis during these time periods.

## Methods

### Study design and participants

The Million Women Study is a population-based prospective study.^15^ In median year 1998 (IQR 1997–1999), women invited for National Health Service (NHS) breast cancer screening at 66 screening centres in England and Scotland were asked to join the study by completing a postal questionnaire about sociodemographic factors, anthropometry, lifestyle factors, and health. The study recruited about half of the eligible women in the participating screening centres: about one in every four UK women born in 1935–50.^15^ The study included a wide range of socioeconomic and educational backgrounds and lifestyles broadly typical of UK women of that generation.^15^ Among survivors, resurvey questionnaires were sent to participants every 3–5 years, assessing changes in these factors and enquiring about other exposures. Information on data access and questionnaires are on the Million Women Study website. Of the women who provided information about cognitive and social activities, we excluded those who by then already had a previous self-report or hospital record of dementia, and a small number with missing information on other activities. Ethical approval was given by the Oxford and Anglia multicentre research ethics committee (97/5/001). Participants gave written consent for recontact and for follow-up through medical records.

### Procedures and outcomes

Questions about participation in various group activities were asked for the first time in median year 2001 (IQR 2001–2003). Women were asked “Do you belong to or participate in any of the following?” with a list of ten activities, to each of which they replied separately. The primary exposures of interest for this analysis were participation in adult education, groups for art, craft, or music, and voluntary work, because these are closest to the cognitive activities commonly recommended to prevent or delay dementia.^1^ Groups for art, craft, or music combined the answers to the separate questions on art or craft groups and on music or singing groups. Identical questions were asked again about 4 years later and, for women who completed both questionnaires, agreement ranged from 85% for voluntary work to 89% for art, craft, or music groups (appendix p 3). Findings for participation in sports clubs, yoga, and dancing are not reported here, because the associations between dementia and physical inactivity in this cohort have been reported recently (appendix p 10).^16^ The question “How many hours in each day do you usually spend reading?” was asked for the first time in median year 2006 (IQR 2006–2006); replies were classified as no reading or any reading, to be consistent with our analyses of the other activities.

All participants were already registered with the NHS at recruitment. Using each individual's unique NHS number and date of birth, they were linked electronically to routinely collected NHS data on hospital admissions (as day cases or inpatients), deaths, and emigrations. Linkage was by NHS Digital in England (where up to 20 diagnoses are coded for every hospital admission) and by the NHS Central Register and Information Services Division in Scotland (where up to six diagnoses are coded for every hospital admission). Hospital diagnoses are coded according to the International Classification of Diseases (ICD)-10.

The main outcome was the first mention of dementia (as ICD-10 codes F00–03 or G30) in a hospital record. We call this dementia detection, even though there might have been previous evidence of dementia in primary care records. To study the difference between the time when dementia was first recorded in primary care and in hospital records, 76 301 participants were also linked to the Clinical Practice Research Datalink (CPRD), which by 2017 included primary care records for 8% of the UK population.^17^, ^18^ For purposes of comparison between CPRD and hospital records, in CPRD, dementia was defined as any of 97 specific Read clinical codes, a code for a drug specifically prescribed for dementia, or both. The median interval between the first mention of dementia, thus defined, in primary care and the first admission to hospital with mention of dementia was 4 (IQR 2–7) years.^16^

### Statistical analysis

Our analyses of cognitive and social activities involved all women who completed the 2001 questionnaire with no previous hospital record of dementia and who were asked about a range of activities, and our analyses of reading included all women who completed the 2006 questionnaire with no previous hospital record of dementia. Cox regression was used to assess hazard ratios (hereafter called risk ratios [RRs] of dementia detection) with their 99% CIs, associating non-participation in various activities at baseline and dementia detection rates during the follow-up periods of 0–4 years, 5–9 years, and 10 or more years. The baseline for participation in adult education, groups for art, craft, or music, and voluntary work was the 2001 questionnaire, and the baseline for reading was the 2006 questionnaire. Time was from the date that the relevant questionnaire was completed until whichever came first of the first hospital record of dementia, death, cessation of NHS registration, or cessation of follow-up (March 31, 2019, in England and Dec 31, 2017, in Scotland).

To ensure that analyses compared like with like, dementia detection RRs were routinely stratified by single year of birth (≤1930, 1931, 1932,…1949, ≥1950), single year of completion of the 2001 questionnaire (or, for the reading analyses, the 2006 questionnaire), and ten regions of residence at baseline (Scotland and the nine English health regions). Analyses also adjusted for the following variables recorded at recruitment (in median year 1998 [IQR 1997–1999]): education (in five categories: tertiary qualifications, secondary qualifications, technical qualifications [nursing, teaching, clerical, or commercial], completed schooling with no qualifications, or did not complete compulsory schooling);^19^ area deprivation (quintiles); frequency of strenuous physical activity (rarely or never, <1 time per week, 1–3 times per week, or >3 times per week); body mass index (<20 kg/m^2^, 20–24·9 kg/m^2^, 25–29·9 kg/m^2^, or ≥30 kg/m^2^); smoking (never, past, currently <10 cigarettes per day, currently 10–19 cigarettes per day, currently ≥20 cigarettes per day, or not current); alcohol consumption (0 units per week, 1–2 units per week, 3–6 units per week, 7–14 units per week, or ≥15 units per week) and use of menopausal hormones (never, past, or current). Some adjustment variables were not recorded in 1998: self-rated health (poor, fair, good, or excellent); currently married or living with a partner (yes or no); paid work (full-time, part-time, or none); and current treatment for depression, diabetes, and high blood pressure (yes or no for each). For these variables, we used information recorded in the 2001 questionnaire, supplemented where necessary by information recorded in the 2006 questionnaire. To keep the numbers analysed constant, the small number of women with missing data for any adjustment variable (<5% for each variable) were assigned to a separate category for that variable. All adjustment measures were self-reported except for area deprivation (Townsend score,^20^ based on the residential postcode in median year 1998). To allow, to some extent, for multiple testing, we used 99% CIs.

For the second decade of follow-up, we sought any heterogeneity between the associations with dementia detection by the age at which the activities had been reported (<60 years, 60–69 years, and ≥70 years), and we assessed the effect of adjustment for each separate potential confounding factor on the associations with dementia detection. Analyses used Stata 15.1, and figures were drawn in R.^21^

### Role of the funding source

The funders of the study had no role in the study design, data collection, data analysis, data interpretation, writing of the report, or the decision to publish. All authors had full access to all of the data in the study and had final responsibility for the decision to submit for publication.

## Results

After the exclusions, 851 307 women answered questions about cognitive or social activities in 2001 and were eligible for these analyses (appendix p 4). They were aged mean 60 years (SD 5) when these activities were recorded. At that time, 102 433 (12%) reported participating in adult education, 110 872 (13%) in groups for art, craft, or music, 161 577 (19%) in voluntary work, and 281 035 (33%) in at least one of these activities. Given the large numbers, many characteristics were significantly associated with participation in these activities (table; appendix pp 5–6). Compared with non-participants, participants tended to be more educated, less likely to report unhealthy factors (eg, smoking or obesity), and less likely to rate their health as poor or fair. After 10 years, only 9591 (1%) of participants had been lost to follow-up, with almost half of these losses being due to emigration; 789 339 (93%) were still alive with no hospital record of dementia and their baseline characteristics were still similar to the entire baseline population (appendix p 7). After exclusions, 655 118 women who answered questions about reading in 2006 were eligible for the analyses of reading (appendix p 4). The mean age of these women was 64 years (SD 5), and the 23 476 (4%) who reported not reading tended to be less educated, to smoke, and to rate their health as poor or fair (table).

**Table:** Characteristics and follow-up of women included in the analyses, by reported participation in cognitive and social activities

		Adult education*		Groups for art, craft, or music*		Voluntary work*		**Reading**†	
		Not participating (n=748 874)	Participating (102 433)	Not participating (n=740 435)	Participating (n=110 872)	Not participating (n=689 730)	Participating (n=161 577)	No reading (n=23 476)	Any reading (631 642)
Mean age at recording activities, years (SD)		60·0 (5·0)	60·2 (5·0)	59·9 (4·9)	60·9 (5·0)	59·8 (4·9)	61·0 (5·0)	63·6 (4·8)	64·4 (4·8)
Variables recorded in median year 1998									
	No educational qualifications	303 225 (41%)	13 408 (13%)	294 819 (41%)	21 814 (20%)	284 474 (42%)	32 159 (20%)	14 332 (63%)	207 381 (33%)
	Most deprived quintile	131 556 (18%)	11 853 (12%)	132 020 (18%)	11 389 (10%)	125 203 (18%)	18 206 (11%)	6138 (26%)	96 642 (15%)
	Rarely or never strenuous exercise	340 237 (47%)	30 706 (30%)	334 225 (46%)	36 718 (34%)	316 955 (47%)	53 988 (34%)	13 555 (60%)	259 754 (42%)
	Current smoker	122 744 (16%)	9151 (9%)	123 592 (17%)	8303 (8%)	117 167 (17%)	14 728 (9%)	5485 (24%)	86 771 (14%)
	Body-mass index ≥30 kg/m^2^	121 475 (17%)	13 778 (14%)	118 538 (17%)	16 715 (16%)	112 072 (17%)	23 181 (15%)	4804 (22%)	96 994 (16%)
	≥15 units of alcohol per week	38 273 (5%)	6357 (6%)	39 183 (5%)	5447 (5%)	36 141 (5%)	8489 (5%)	1186 (5%)	34 409 (5%)
	Ever user of menopausal hormones	375 734 (51%)	54 356 (53%)	373 572 (51%)	56 518 (51%)	350 423 (51%)	79 667 (50%)	11 569 (50%)	324 934 (52%)
Variables recorded in median year 2001									
	Poor or fair self-rated health‡	180 333 (25%)	17 310 (17%)	177 019 (25%)	20 624 (19%)	169 075 (25%)	28 568 (18%)	8530 (37%)	132 178 (21%)
	Not married or no partner‡	140 463 (19%)	23 932 (24%)	141 077 (19%)	23 318 (21%)	127 892 (19%)	36 503 (23%)	5626 (24%)	129 561 (21%)
	Not in paid work‡	412 442 (55%)	58 623 (57%)	400 468 (54%)	70 597 (64%)	364 421 (53%)	106 644 (66%)	13 825 (59%)	359 740 (57%)
	Treated for high blood pressure	172 807 (23%)	19 505 (19%)	168 821 (23%)	23 491 (21%)	158 099 (23%)	34 213 (21%)	4207 (18%)	115 408 (18%)
	Treated for diabetes	26 629 (4%)	2441 (2%)	25 871 (3%)	3199 (3%)	24 331 (4%)	4739 (3%)	853 (4%)	15 456 (2%)
	Treated for depression	51 031 (7%)	6871 (7%)	50 184 (7%)	7718 (7%)	47 480 (7%)	10 422 (6%)	1718 (7%)	33 812 (5%)
Follow up for dementia									
	Person-years, 1000s	11 805	1643	11 683	1765	10 880	2569	278	8626
	Women diagnosed with dementia	27 924 (4%)	3263 (3%)	27 047 (4%)	4140 (4%)	24 748 (4%)	6439 (4%)	1032 (4%)	19 983 (3%)
	Mean (SD) age at dementia diagnosis, years	77·7 (5·6)	78·4 (5·7)	77·7 (5·6)	78·7 (5·4)	77·6 (5·6)	78·6 (5·5)	76·4 (5·8)	78·4 (5·3)

Data are mean (SD), n (%), or n.

* These activities were recorded in median year 2001.

† Reading was recorded in median year 2006.

‡ For analyses of reading, information on self-rated health, marital status, or paid work was not reported in 2001 by 18% of the women, and the missing information was supplemented by information reported in median year 2006.

Follow-up after the 2001 survey was for a mean of 16 years (SD 3), during which all but 85 933 (10%) of 851 307 participants had at least one hospital admission and 31 187 (4%) had at least one hospital admission with mention of dementia. Of these 31 187 women, 21 142 (68%) had more than one hospital admission, most of whom (18 365 [87%]) had dementia mentioned again at a subsequent admission. Furthermore, of these 31 187 women, 848 (3%) had hospital admissions with dementia mentioned for the first time during years 0–4, 4703 (15%) during years 5–9, and 25 636 (82%) during 10 or more years, the latter at mean age 79 years (SD 5).

**Figure 1:**
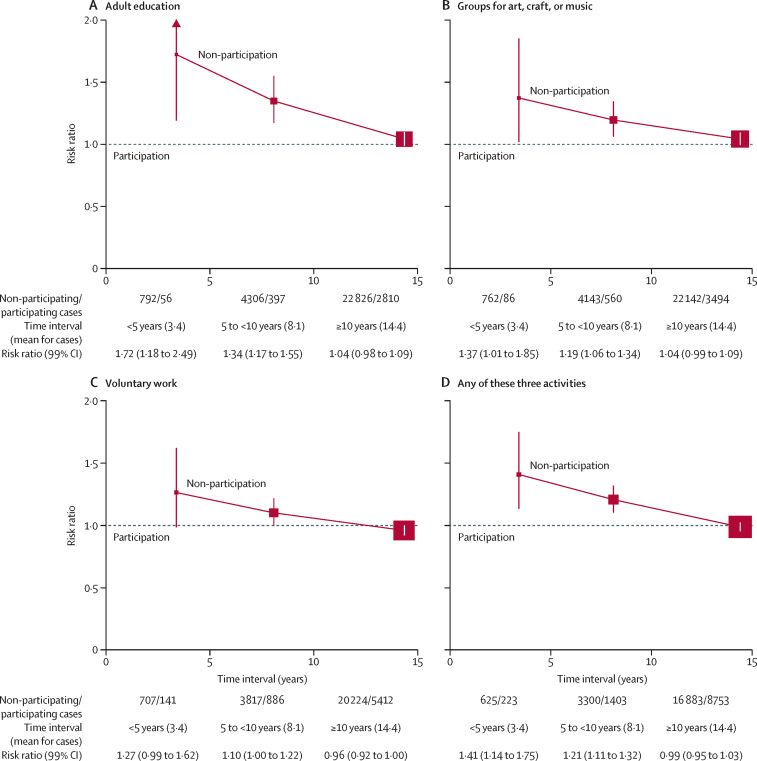
Relevance of the time interval between the recording of dementia and recording of participation in various cognitive and social activities, including adult education (A), groups for art, craft, or music (B), voluntary work (C), or any of those three activities (D) Error bars show 99% CI. The size of each red square is proportional to the amount of statistical information. The values of the risk ratio in these analyses assess the associations between dementia having been recorded during a hospital admission for the first time and non-participation (*vs* participation) in various activities having been reported 0–4, 5–9, and 10 or more years earlier. The strengths of the associations for different time intervals can help assess whether, particularly with the shorter time intervals, there are only the expected effects of dementia or incipient dementia on non-participation.

The dementia detection RRs during follow-up 0–4, 5–9, and 10 or more years for non-participation versus participation in adult education, groups for art, craft, or music, and voluntary work are shown in figure 1, and in each case the findings were similar. Non-participation was associated with dementia incidence strongly during years 0–4, less strongly during years 5–9, and hardly at all during the second decade. Thus, non-participation in adult education was associated with dementia detection only during the first decade, whereas during the second decade of follow-up (mean 14 years [SD 2] for cases), little or no association remained (RR 1·04, 99% CI 0·98–1·09; figure 1A). Likewise, non-participation in groups for art, craft, or music showed little or no association during the second decade of follow-up (RR 1·04, 0·99–1·09; figure 1B), as did non-participation in voluntary work (RR 0·96, 0·92–1·00; figure 1C). Finally, the RR for non-participation in any of these three types of activity was null (RR 0·99, 0·95–1·03) during the second decade of follow-up (figure 1D). For each activity, the adjustment factors that most affected the minimally adjusted associations (ie, with adjustment just for age and region of residence) during the second decade were educational qualifications and self-rated health (appendix p 8). During the follow-up period of 10 or more years, there was little dependence of these RRs on the age at which these activities were reported, although confidence intervals were wide (appendix p 9). For example, the RRs associated with non-participation in any of these activities were 0·96 (99% CI 0·88–1·05) for women reporting these activities in their 50s, 1·00 (0·96–1·04) in their 60s, and 1·01 (0·90–1·13) in their 70s (appendix p 9).

**Figure 2:**
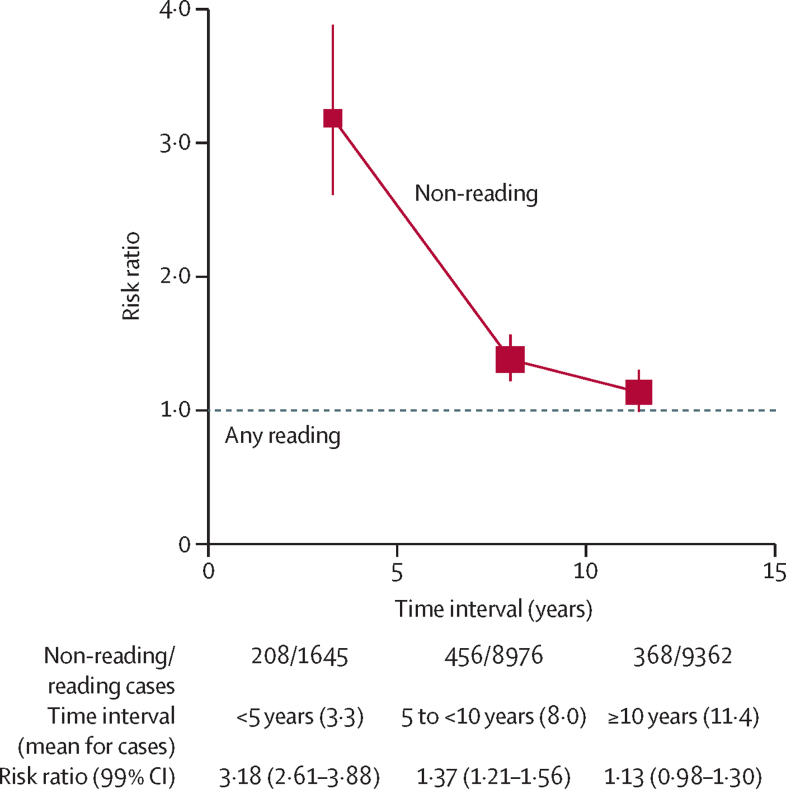
Relevance of the time interval between the recording of dementia and recording of reading Error bars show 99% CI. The strengths of the associations for different time intervals can help assess whether, particularly with the shorter time intervals, there are only the expected effects of dementia or incipient dementia on non-reading.

Time spent reading was first reported about 4 years later than group activities had been reported, so mean follow-up was shorter at only 12 years (SD 2). During this follow-up, 21 015 (3%) of 655 118 women with information on reading had a first hospital record of dementia, of which 1853 (9%) were during years 0–4, 9432 (45%) were during years 5–9, and 9730 (46%) were during the second decade of follow-up of the question about reading, the latter group of onsets occuring at mean age 78 years (SD 5). Figure 2 shows dementia detection RRs by follow-up period among women who reported not reading compared to any reading. The RR was 3·18 (99% CI 2·61–3·88) during years 0–4 of follow-up, but declined substantially with follow-up duration and was 1·13 (0·98–1·30) the second decade of follow-up of the question about reading. Moreover, the latter group of onsets occurred only just after year 10, because the mean duration of follow-up for cases in this group was only 11 years (SD 1).

## Discussion

The UK Million Women prospective study is large with a long follow-up, averaging 16 years. Dementia increases steeply with age, so although there were 5551 first hospital records of dementia during the first decade of follow-up, there were 25 636 during the second. This allows reliable analyses of dementia detection risks not only (as in other studies) during the first decade, but also (unlike in other studies) during the second decade. Non-participation in cognitive or social activities was (as in other studies) strongly associated with dementia detection risk during the first 5 years of follow-up and moderately associated with dementia detection risk during follow-up years 5–9, but had little association with dementia detection risk during the second decade of follow-up. The inverse associations of cognitive and social activities with dementia risk during the first decade, together with the approximately null associations during the second decade, are similar to the findings for other factors, such as physical inactivity.^13^, ^16^ In particular, a recent report^16^ from the present study, using similar methods, has shown that physical inactivity likewise has no independent association with dementia rates during the second decade of follow-up. Taken together, these findings provide strong evidence that, several years before dementia is diagnosed clinically, there is a progressive reduction in participation in various mental and physical activities, and that the activities themselves have little or no causal relevance to the incidence of dementia. In other words, the mental and physical activities assessed by our questions have, in themselves, no material protective effect against the development of clinically apparent dementia.

Most previously published prospective studies of such associations had follow-up periods shorter than 10 years^5^, ^22^, ^23^, ^24^, ^25^, ^26^ and our short-term findings (ie, during the first decade) are consistent with theirs. Five studies of cognitive and social factors had some follow-up beyond the first decade,^6^, ^7^, ^8^, ^9^, ^10^ of which only two^9^, ^10^ reported the associations separately during short-term and during long-term follow-up. The findings of these two studies, although based on small numbers, are consistent with the present findings: the Betula prospective study in Sweden,^10^ with 357 incident cases over 15 years of follow-up, split its results into three 5-year periods and found that dementia incidence was associated with non-participation in social activity only in the first period; and the Prospective Population Study of Women in Sweden,^9^ with 194 incident cases over 44 years of follow-up,^9^ found that this association was weakened when the first 20 years of follow-up were excluded. In the present study, with 31 187 incident cases, most women were in their 60s at the time of reporting their activities, and the lack of any material association with dementia incidence during the second decade of follow-up appeared similar for those who were in their 50s, 60s, and 70s at the time of reporting. This finding is strong evidence against the hypothesis that an absence of cognitive activity in middle age or in old age causes dementia.^2^

Mechanisms proposed to explain how participation in cognitive and social activities might causally affect dementia incidence include the enhancement of cognitive reserve by using more efficient neuronal networks or alternative networks,^2^, ^27^, ^28^ and the ameliorating effects of social contact,^2^, ^29^ but neither is supported by our findings during the second decade of follow-up. Minimally adjusted associations during the second decade of follow-up were generally attenuated more by adjustment for education than by adjustment for other factors. Whether or not education itself has any protective effect, it could help those developing dementia to delay diagnosis of their condition.^22^

The strengths of this study include virtually complete long-term follow-up (only 1% lost after 10 years) and adjustment for many potential confounders. The prospective design limits bias in recall of the activities. With 31 187 women having dementia first recorded during follow-up, including 25 636 in the second decade, there were sufficient numbers to show reliably that apparently strong short-term associations with cognitive or social activities virtually disappear within a decade. The large numbers of cases also allowed us to examine separately the associations with adult education, groups for art, craft, or music, or voluntary groups, showing in each case the disappearance of any material association by the second decade of follow-up. Reading showed a similar pattern, and longer follow-up will allow even clearer investigation. The questions about group activities were simple and did not include the duration and frequency of each activity, but they are validated by being strongly predictive of dementia onset rates during follow-up years 0–4, and by the good agreement between the same questions 4 years apart. Our questions examined cognitive and social activities that closely represent the types recommended for dementia prevention,^1^ although there is no agreed definition or measure of such activities.^5^

The main limitation, as in most other studies, is uncertainty as to when dementia starts developing. Some years can pass between the time when behaviour is first affected, when dementia is first recorded in primary care, when dementia is first diagnosed after careful clinical examination, and when dementia is first recorded in hospital. Moreover, not all women with dementia will have been admitted to hospital, and some women with dementia must have been admitted to hospital without this being recorded. Encouragingly, a study^30^ comparing NHS hospital diagnoses and expert clinical adjudication showed a positive predictive value of 87% for hospital diagnoses of dementia (albeit with somewhat lower values for the separate endpoints of Alzheimer's disease and vascular dementia, which were not investigated separately in the present analyses).^30^ In addition, for a randomly selected subset of 1000 women in this cohort with no hospital record of dementia, enquiry to their primary care physicians identified only one with a primary care record of dementia. Moreover, in the 76 301 women for whom primary care records were also available, about 95% of the hospital diagnoses of dementia could be confirmed in primary care records,^17^ although the median time from a first mention of dementia in primary care to its first mention in a hospital record was 4 years.^17^ Nevertheless, the large majority of women categorised in hospital records as having dementia and the large majority categorised as not having dementia are likely to have been correctly classified. Any misclassification would be expected to dilute real associations slightly, but not to have a substantial differential effect on associations during different follow-up periods. Although the reasons that women were admitted to hospital might change over time, any changes over each 5-year period are unlikely to be great enough to produce the large differences in the RRs seen here. Another limitation is that the study was confined to women.

When dementia has reached the point that it is clinically apparent, its causes can be understood only by looking backwards in studies that follow participants forwards (to paraphrase Kierkegaard). Our findings, and those of other studies with sufficiently long follow-up,^9^, ^10^, ^13^, ^16^ show that long before dementia is diagnosed, there is a progressive reduction in various mental and physical activities, chiefly because its gradual onset causes inactivity, not because inactivity causes dementia. Current UK and international guidelines recommend cognitive and physical activities to prevent dementia,^1^, ^2^, ^3^, ^4^ but in the light of the available evidence, these guidelines should be reconsidered.

For the **Million Women Study Data Access Policy** see millionwomenstudy.org/data_access

## Data sharing

Anonymised data used here are available to any qualified researcher upon request to the investigators (addressed to mws.access@ndph.ox.ac.uk) and to the providers of follow-up data (eg, NHS Digital). The Million Women Study Data Access Policy can be viewed online.
