# Supplementary material for: Cognitive and social activities and long-term dementia risk: the prospective UK Million Women Study
Source: Lancet Public Health. 2021 Jan 28;6(2):e116–23. doi: 10.1016/S2468-2667(20)30284-X (PMC7848753; doi:10.1016/S2468-2667(20)30284-X)
Supplement: Supplementary appendix [file mmc1.pdf]

THE LANCET
Public Health

Supplementary appendix

This appendix formed part of the original submission and has been peer reviewed. We post it as supplied by the authors.

Supplement to: Floud S, Balkwill A, Sweetland S, et al. Cognitive and social activities and long-term dementia risk: the prospective UK Million Women Study. *Lancet Public Health* 2021; **6:** e116–23.

This blank page is inserted to rectify the appendix page numbering during the Lancet Public Health editorial process

CONTENTS

Supplementary Figures and Tables	Page
**eTable 1.** Agreement of reported participation in cognitive and social activities in two questionnaires completed 4 years apart (n=569,357)	**3**
**eFigure 1.** Flow chart of women included in the analyses of cognitive and social activities (out of 1.3 million recruited in median year 1998)	**4**
**eTable 2.** Characteristics at recruitment (in 1998) for women included in the analyses, by participation in certain cognitive and social activities, showing each category of covariate	**5-6**
**eTable 3.** Characteristics at recruitment (in 1998) for women included in the analyses who were still alive after 10 years of follow-up and with no previous dementia detected, by participation in certain cognitive and social activities	**7**
**eTable 4.** Dementia detection risk ratios during the second decade of follow-up after various activities were recorded, comparing non-participation vs participation in them: effect of adjustment by various factors	**8**
**eTable 5.** Dementia detection risk ratios during the second decade of follow-up after various activities were recorded, comparing non-participation vs participation in them: effect of decade of age at recording	**9**
**eFigure 2.** Million Women Study results for PHYSICAL inactivity: Dementia detection rate ratios, inactive vs active, during various time periods after recording PHYSICAL activity (*Neurology* 2020; 94: e123–32)	**10**
**Acknowledgements** Membership of the Million Women Study Advisory Committee; Million Women Study coordinating centre staff; NHS Breast Cancer Screening Programme collaborating centres	**11**

eTable 1. Agreement of reported participation in cognitive and social activities in two questionnaires completed 4 years apart (n=569,357)

Cognitive/social activity	Agreement
Adult education	86%
Art/craft/music	89%
Voluntary work	85%

eFigure 1. Flow chart of women included in the analyses of cognitive and social activities (out of 1.3 million recruited in median year 1998)

A. For analyses of 3 cognitive/social activities & risk of dementia

B. For analyses of reading & risk of dementia

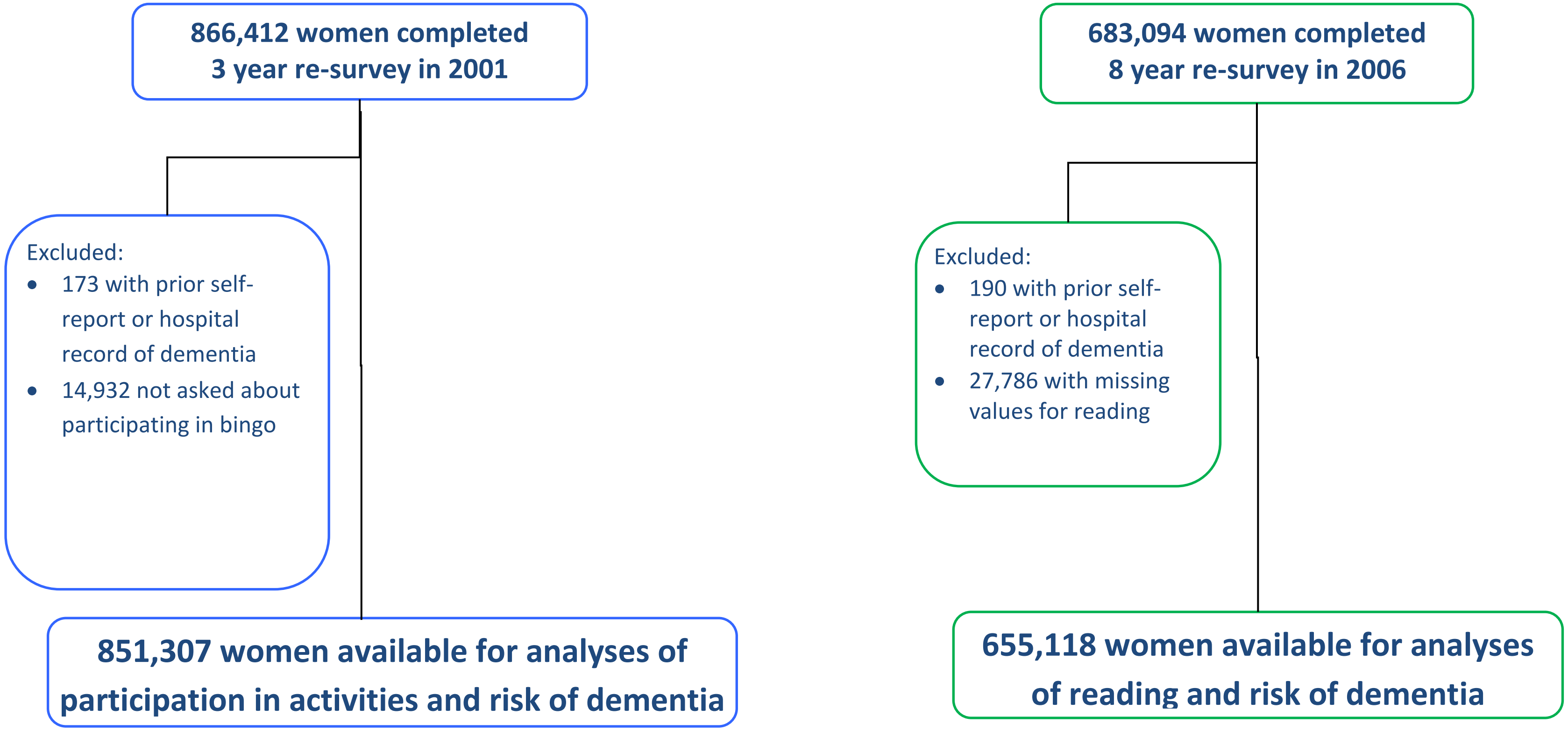

eTable 2. Characteristics at recruitment (in 1998) for women included in the analyses, by participation in certain cognitive and social activities, showing each category of covariate

	Adult education (baseline for these analyses was median year 2001		Art/craft/music groups (baseline for these analyses was median year 2001)		Voluntary work (baseline for these analyses was median year 2001)		Reading (baseline for these analyses was median year 2006)	
	Not participating	Participating	Not participating	Participating	Not participating	Participating	No reading	Any reading
Women, n	748,874	102,433	740,435	110,872	689,730	161,577	23,476	631,642
Mean age at recording activities, years (SD)	60.0 (5.0)	60.2 (5.0)	59.9 (4.9)	60.9 (5.0)	59.8 (4.9)	61.0 (5.0)	63.6 (4.8)	64.4 (4.8)
**Variables recorded in median year 1998**								
Education, %								
Tertiary qualifications	13	34	14	29	13	27	6	18
Secondary qualifications	28	37	28	34	28	36	17	31
Technical qualifications	17	16	17	17	17	17	15	17
Completed compulsory schooling with no qualifications	39	13	39	19	40	19	59	32
Did not complete compulsory schooling	2	1	2	1	2	1	4	1
Deprivation, %								
Q1 (least deprived)	21	26	21	26	21	25	16	23
Q2	21	24	21	24	21	24	18	22
Q3	20	21	20	22	20	22	19	21
Q4	20	18	20	18	20	18	21	19
Q5 (most deprived)	18	12	18	10	18	11	26	15
Strenuous exercise, %								
Rarely/never	47	30	46	34	47	34	60	42
<once a week	13	16	13	16	13	16	9	14
1-3 times a week	34	46	34	43	34	43	24	37
>3 times a week	6	7	6	7	6	7	7	6
Smoking, %								
Never	51	57	50	61	50	60	44	54
Past	28	30	28	27	28	27	27	28
Current <10 cigarettes per day	4	3	4	3	4	3	5	4

Current 10-19 cigarettes per day	8	4	8	3	8	4	11	7
Current 20+ cigarettes per day	4	2	4	2	4	2	7	3
Body mass index kg/m^2, %								
<20	4	4	4	4	4	4	4	4
20-24.9	44	49	44	46	44	47	39	45
25-29.9	36	33	35	35	35	35	35	35
30+	17	14	17	16	17	15	22	16
Alcohol units/week, %								
0	22	14	22	17	22	18	34	19
1-2	31	31	31	33	31	32	29	32
3-6	22	26	22	25	22	25	17	24
7-14	19	23	19	20	19	20	15	20
15+	5	6	5	5	5	5	5	5
Use of menopausal hormones, %								
Never	49	47	49	49	49	50	50	48
Past	17	18	17	18	17	17	17	17
Current	34	36	34	33	34	32	33	35
**Variables recorded in median year 2001**								
Self-rated health, % ‡								
Poor	3	2	3	2	3	2	7	2
Fair	22	16	22	17	22	17	30	19
Good	59	60	59	60	58	61	51	61
Excellent	16	22	17	21	16	21	12	18
Not married/partner, % ‡	19	24	19	21	19	23	24	21
Paid work, % ‡								
Full-time	19	17	20	13	21	11	17	18
Part-time	24	25	25	23	25	22	23	25
None	55	57	54	64	53	66	59	57
High blood pressure, %	23	19	23	21	23	21	18	18
Diabetes, %	4	2	3	3	4	3	4	2
Depression, %	7	7	7	7	7	6	7	5

‡ For analyses of reading, information on self-rated health, marital status or paid work was not reported in 2001 by 18% of the women, and so the missing information was supplemented by information reported in median year 2006

eTable 3. Characteristics at recruitment (in 1998) for women included in the analyses who were still alive after 10 years of follow-up and with no previous dementia detected, by participation in certain cognitive and social activities

	Adult education (baseline for these analyses was median year 2001)		Art/craft/music groups (baseline for these analyses was median year 2001)		Voluntary work (baseline for these analyses was median year 2001)		Reading (baseline for these analyses was median year 2006)	
	Not participating	Participating	Not participating	Participating	Not participating	Participating	No reading	Any reading
Women, n	692,903	96,436	685,490	103,849	638,275	151,064	19,997	569,840
Mean age at recording activities, years (SD)	59.9 (5)	60.0 (5)	59.7 (5)	60.8 (5)	59.7 (5)	60.8 (5)	63.1 (5)	64.1 (5)
**Variables recorded in median year 1998**								
No educational qualifications, %	41	13	40	20	42	20	62	32
Most deprived fifth, %	17	11	17	10	18	11	25	15
Rarely/never strenuous exercise, %	46	30	46	33	47	34	58	41
Current smoker, %	16	9	16	7	16	9	21	13
Body mass index >=30 kg/m^2, %	17	14	16	15	17	15	21	15
Alcohol 15+ units/week, %	5	6	5	5	5	5	5	5
Ever user of menopausal hormones, %	51	54	51	52	52	50	51	52
**Variables recorded in median year 2001**								
Poor/fair self-rated health, %‡	23	16	23	19	24	17	33	19
Not married/partner, %‡	19	23	19	21	18	22	23	20
Not in paid work, %‡	54	57	53	63	52	65	55	55
Treated for high blood pressure, %	22	19	22	21	22	21	17	18
Treated for diabetes, %	3	2	3	3	3	3	3	2
Treated for depression, %	7	7	7	7	7	6	7	5
**Follow up for dementia:**								
Person-years, 1000s	11,477	1,608	11,361	1,724	10,579	2,506	254	8,100
Women diagnosed with dementia, n	22,826	2,810	22,142	3,494	20,224	5,412	368	9,362
Mean (SD) age at dementia diagnosis, years	78.7 (5)	79.2 (5)	78.6 (5)	79.5 (5)	78.6 (5)	79.4 (5)	79.1 (5)	80.1 (5)

‡ For analyses of reading, information on self-rated health, marital status or paid work was not reported in 2001 by 18% of the women, and so the missing information was supplemented by information reported in median year 2006

eTable 4. Dementia detection risk ratios during the second decade of follow-up after various activities were recorded, comparing non-participation vs participation in them: effect of adjustment by various factors

	Adult Education<br>RR (99% CI)	Art/craft/music groups<br>RR (99% CI)	Voluntary work<br>RR (99% CI)	Reading<br>RR (99% CI)
**Adjusted by age and region only**	**1.20 (1.14-1.27)**	**1.16 (1.11-1.22)**	**1.09 (1.05-1.13)**	**1.38 (1.20-1.58)**
**Additionally adjusted separately by:**				
Education	1.09 (1.03-1.15)	1.08 (1.03-1.14)	1.01 (0.97-1.05)	1.29 (1.12-1.48)
Area deprivation	1.17 (1.11-1.24)	1.13 (1.08-1.18)	1.06 (1.02-1.10)	1.33 (1.16-1.52)
Strenuous exercise	1.15 (1.10-1.22)	1.13 (1.07-1.18)	1.05 (1.01-1.10)	1.33 (1.16-1.53)
Body mass index	1.19 (1.13-1.25)	1.16 (1.10-1.21)	1.08 (1.04-1.12)	1.35 (1.18-1.55)
Smoking	1.17 (1.11-1.23)	1.12 (1.07-1.17)	1.05 (1.01-1.10)	1.32 (1.15-1.52)
Alcohol consumption	1.16 (1.10-1.22)	1.14 (1.08-1.19)	1.06 (1.02-1.11)	1.32 (1.15-1.52)
Menopausal hormone use	1.20 (1.14-1.27)	1.16 (1.11-1.22)	1.09 (1.04-1.13)	1.38 (1.20-1.58)
Self-rated health	1.13 (1.07-1.19)	1.12 (1.06-1.17)	1.02 (0.98-1.07)	1.23 (1.07-1.41)
Marital status	1.22 (1.15-1.28)	1.17 (1.11-1.22)	1.10 (1.05-1.14)	1.36 (1.19-1.56)
Paid work	1.21 (1.14-1.27)	1.17 (1.12-1.23)	1.10 (1.06-1.15)	1.36 (1.19-1.56)
High blood pressure	1.19 (1.13-1.25)	1.15 (1.10-1.21)	1.08 (1.04-1.12)	1.38 (1.20-1.58)
Diabetes	1.19 (1.13-1.25)	1.15 (1.10-1.21)	1.08 (1.04-1.12)	1.36 (1.19-1.56)
Depression	1.20 (1.14-1.26)	1.16 (1.11-1.22)	1.08 (1.05-1.13)	1.36 (1.19-1.56)
**Fully adjusted for all the above**	**1.04 (0.98-1.09)**	**1.04 (0.99-1.09)**	**0.96 (0.92-1.00)**	**1.13 (0.98-1.30)**

eTable 5. Dementia detection risk ratios during the second decade of follow-up after various activities were recorded, comparing non-participation vs participation in them: effect of age at recording

	**Decade of age at recording participation in the activity**		
	**During women's 50s**	**During women's 60s**	**During women's 70s**
**Adult education**	0.97 (0.85-1.10)	1.07 (1.00-1.14)	1.00 (0.85-1.18)
**Art/craft/music groups**	1.00 (0.88-1.13)	1.04 (0.98-1.10)	1.08 (0.94-1.25)
**Voluntary work**	0.92 (0.83-1.02)	0.97 (0.92-1.02)	1.01 (0.89-1.15)
**Any of above activities**	0.96 (0.88-1.05	1.00 (0.96-1.04)	1.01 (0.90-1.13)
**Reading**	1.14 (0.69-1.89)	1.04 (0.86-1.26)	1.26 (1.01-1.59)

eFigure 2. Million Women Study results for PHYSICAL inactivity: Dementia detection rate ratios, inactive vs active, during various time periods after recording PHYSICAL activity (*Neurology* 2020; 94: e123–32)

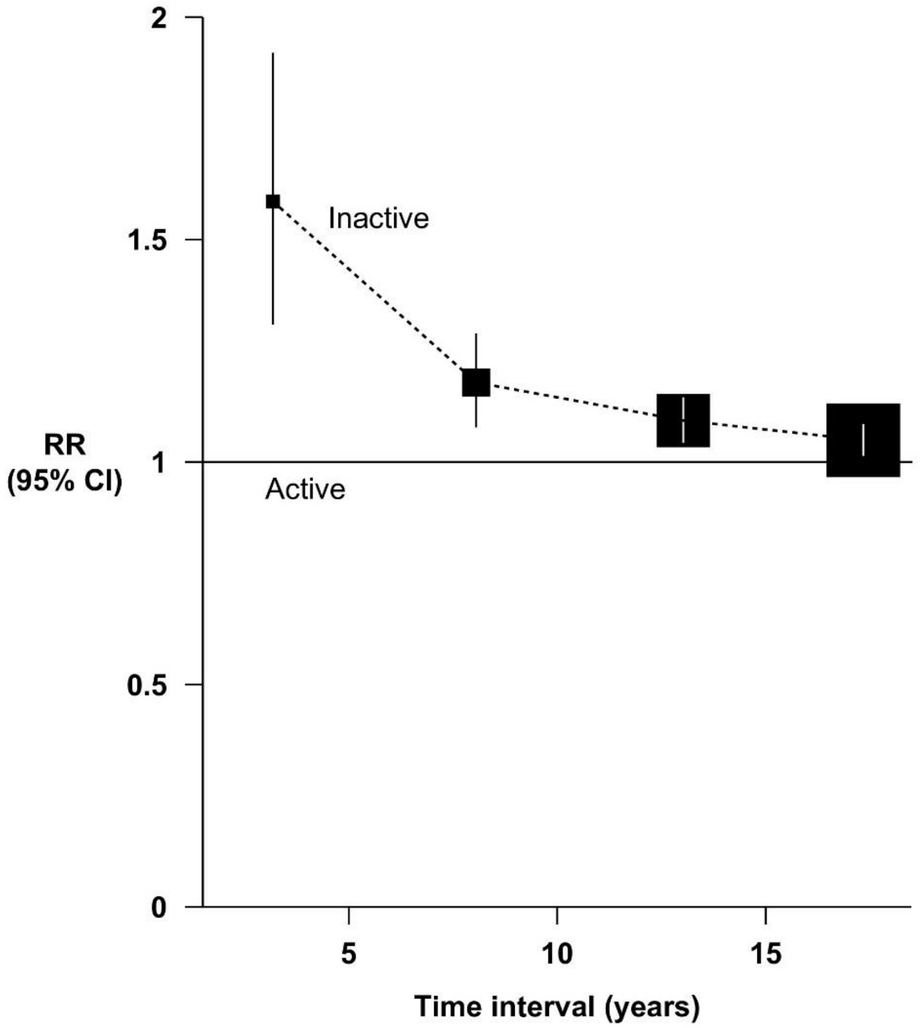

**Inactive/active cases**	196/282	824/1586	3054/6320	5843/12852
**Time interval (mean for cases)**	<5 years (3.2)	5-<10 years (8.1)	10-<15 years (13.0)	15+ years (17.3)
**RR (95% CI)**	1.59 (1.31, 1.92)	1.18 (1.08, 1.29)	1.09 (1.05, 1.14)	1.05 (1.02, 1.08)

Stratified by year of birth and year reporting exposure; and adjusted for region of residence, educational qualifications, area deprivation, height, smoking, alcohol consumption, use of menopausal hormones and BMI.

Acknowledgements

Membership of the Million Women Study Advisory Committee

Emily Banks, Valerie Beral, Lucy Carpenter, Carol Dezateux (chair), Sarah Floud, Jane Green, Julietta Patnick, Richard Peto, Gillian Reeves, Cathie Sudlow.

Million Women Study coordinating centre staff

Simon Abbott, Rupert Alison, Krys Baker, Angela Balkwill, Isobel Barnes, Valerie Beral, Judith Black, Roger Blanks, Anna Brown, Andrew Chadwick, Dave Ewart, Sarah Floud, Kezia Gaitskell, Toral Gathani, Laura Gerrard, Adrian Goodill, Jane Green, Jane Henderson, Carol Hermon, Darren Hogg, Isobel Lingard, Sau Wan Kan, Nicky Langston, Kirstin Pirie, Gillian Reeves, Keith Shaw, Emma Sherman, Helena Strange, Siân Sweetland, Ruth Travis, Lyndsey Trickett, Clare Wotton, Owen Yang, Heather Young.

NHS Breast Cancer Screening Programme collaborating centres

The following NHS Breast Screening Centres took part in the recruitment and breast screening follow-up for the Million Women Study:

Avon, Aylesbury, Barnsley, Basingstoke, Bedfordshire & Hertfordshire, Cambridge and Huntingdon, Chelmsford and Colchester, Chester, Cornwall, Crewe, Cumbria, Doncaster, Dorset, East Berkshire, East Cheshire, East Devon, East of Scotland, East Suffolk, East Sussex, Gateshead, Gloucestershire, Great Yarmouth, Hereford and Worcester, Kent, Kings Lynn, Leicestershire, Liverpool, Manchester, Milton Keynes, Newcastle, North Birmingham, North East Scotland, North Lancashire, North Middlesex, North Nottingham, North of Scotland, North Tees, North Yorkshire, Nottingham, Oxford, Portsmouth, Rotherham, Sheffield, Shropshire, Somerset, South Birmingham, South East Scotland, South East Staffordshire, South Derbyshire, South Essex, South Lancashire, South West Scotland, Surrey, Warrington Halton St Helens and Knowsley, Warwickshire Solihull and Coventry, West Berkshire, West Devon, West London, West Suffolk, West Sussex, Wiltshire, Winchester, Wirral, Wycombe.
